# Novel Use of Chimeric Serratus Anterior Fascia–Latissimus Dorsi Flap for Composite Cranial Defect

**DOI:** 10.1055/a-2778-4044

**Published:** 2026-03-05

**Authors:** James Antongiovanni, Nikhil Shah, Chizoba A. Mosieri, Sarah Struble, Hoyune E. Cho, Medha Vallurupalli, Gabrielle Labove, Miles J. Pfaff

**Affiliations:** 1 Department of Plastic Surgery, University of California, Irvine, School of Medicine, Orange, California, United States; 2 Washington State University, Elson S. Floyd College of Medicine, Spokane, Washington, United States; 3 Louisiana State University Health Shreveport School of Medicine, Shreveport, Los Angeles, United States; 4 University of Southern California, Keck School of Medicine, Los Angeles, California, United States; 5 Children's Hospital of Orange County, Orange, California, United States

**Keywords:** cranioplasty, chimeric free flap, calvarial reconstruction, craniotomy, multilayer scalp reconstruction

## Abstract

Effective soft tissue coverage is essential for minimizing complications in large cranial defects. This case report describes the successful application of a chimeric serratus anterior fascia–latissimus dorsi (SAFLD) flap for single-stage coverage of a large cranial defect. This technique is compared to the current literature. A 69-year-old female with a history of glioblastoma and a rapidly growing scalp squamous cell carcinoma underwent en bloc resection and single-stage reconstruction. After cranioplasty, a large defect was covered with an SAFLD flap, ensuring multilayered vascularized soft tissue coverage. Recovery was uneventful, and a 1-year follow-up demonstrated good cranial morphology. This case demonstrates the successful application of a chimeric free flap for dual-layer coverage in cranial reconstruction, potentially reducing complications associated with single-stage repairs and improving patient outcomes. A literature review demonstrating various case applications of this flap is also presented.

## Introduction

The goal of immediate soft tissue coverage for large composite cranial defects is to minimize wound complications by providing robust, vascularized soft tissue coverage over the cranioplasty site. Large defects involving the scalp and calvarial bone are often repaired with cranioplasty and immediate soft tissue coverage using free tissue transfer. 1 2 Factors such as multiple neurosurgical operations and radiation-related wound bed changes can complicate primary scalp repair. 2 This has led to a growing interest in single-stage reconstruction of large composite defects with cranioplasty and free soft tissue coverage. 1

Multiple methods of free tissue transfer have been described for scalp reconstruction, with the latissimus dorsi muscle flap most frequently utilized. 3 More recently, chimeric flaps based on the robust subscapular system have been described to aid in the reconstruction of both large scalp defects and composite defects involving bone. This has included harvest of a myo-osseous serratus anterior flap with ribs for vascularized bone coverage, as well as chimeric flaps with the serratus anterior muscle placed adjacent to the latissimus dorsi muscle for large defects. 4 5

A commonly reported complication of single-stage reconstruction for composite defects is flap atrophy and late-stage cranioplasty or hardware exposure. 6 7 Utilizing multiple layers of closure is thought to mitigate such breakdown. 8 In this context, we present a case that demonstrates the potential of a chimeric serratus anterior fascia–latissimus dorsi (SAFLD) flap providing multilayered, vascularized soft tissue coverage over a cranioplasty site during single-stage reconstruction of a large composite cranial defect. Further, a review of the literature is presented on scalp reconstruction using the latissimus flap and variations of the chimeric latissimus flap.

## Case

### Presentation and Operative Procedure

A 69-year-old female with a history of glioblastoma multiforme (GBM) was diagnosed with a rapidly growing squamous cell carcinoma of the left parietal scalp with calvarial extension. She had undergone two craniotomies and surgical resections for her GBM approximately 2 years prior to presentation. This was followed by adjuvant chemoradiation, with her last radiation treatment 26 months prior to presentation and her final round of chemotherapy 21 months prior. She continued to intermittently use electrical field therapy. After shared decision-making with the patient and multiple medical and surgical specialties, including neurosurgery, surgical oncology, and plastic surgery, the decision was made to proceed with resection and single-stage reconstruction.

**Fig. 1:**
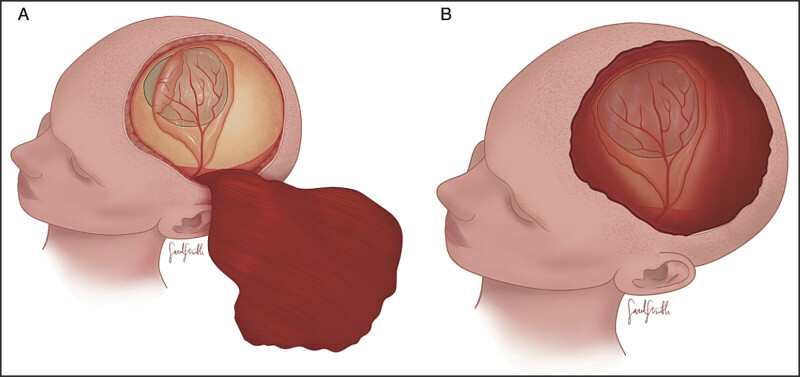
Perioperative (A– **C** ) and postoperative ( **D, E** ) photographs. ( **A** ) Perioperative soft tissue defect (measuring 13 cm × 18 cm) and cranioplasty (measuring 7 cm × 5 cm). ( **B** ) SAFLD free flap, showing the serratus anterior (SA) fascia and latissimus dorsi (LD) muscles. ( **C** ) SAFLD flap in situ demonstrating perfusion via indocyanine green imaging (SPY Fluorescence Imaging; Stryker). ( **D, E** ) Approximately 1-year follow-up. SAFLD, serratus anterior fascia–latissimus dorsi.

In the operating room, the patient was positioned in the right lateral decubitus position, then prepped and draped in the standard sterile fashion. The surgical oncology team first performed an en bloc excision of the lesion with 1 cm margins, including the underlying periosteum. Several frozen sections were taken from peripheral margins, all of which were negative for malignancy. The neurosurgical team then performed a full-thickness craniectomy with 1 cm margins of bone. A collagen matrix was placed over the exposed dura, followed by a methyl methacrylate cranioplasty. The soft tissue defect measured approximately 13 cm × 18 cm, and the cranioplasty measured 7 cm × 5 cm ( Fig. 1A ). It was decided to proceed with a chimeric free flap-based reconstruction using the left latissimus dorsi muscle and the left serratus anterior muscle fascia based on the thoracodorsal pedicle. This was harvested through a curvilinear left midaxillary incision. The serratus branch of the thoracodorsal vessels was preserved, and the serratus anterior muscle fascia was elevated off the muscle ( Fig. 1B ). Care was taken to preserve innervation to the serratus anterior muscle. Indocyanine green angiography was used to confirm adequate perfusion of both flaps prior to harvest, showing that both the serratus fascial flap and the latissimus muscle flap were perfused ( Fig. 1C ).

**Fig. 2:**
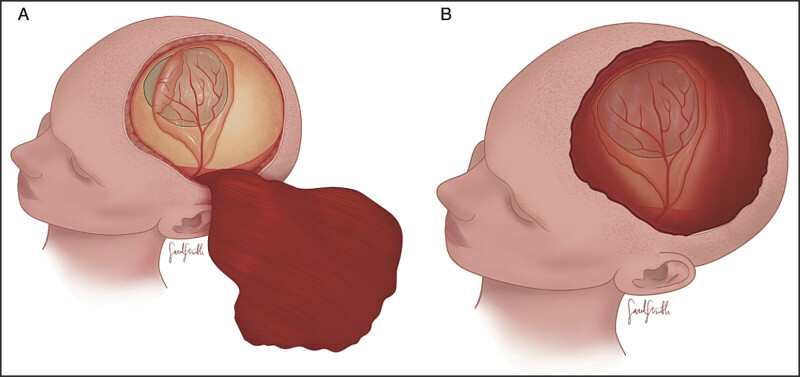
Illustration of the SAFLD flap inset. ( **A** ) Inset of serratus anterior fascia over cranioplasty. ( **B** ) Final flap inset with the latissimus muscle overlaying the serratus anterior fascia and remaining soft tissue defect. SAFLD, serratus anterior fascia–latissimus dorsi.

The left superficial temporal artery was identified, but an adequate vein was not found in the preauricular region. The left external jugular vein (EJV) was then identified and used for anastomosis. To avoid pedicle stretch, a lesser saphenous vein graft was harvested from the left leg to provide additional length for venous anastomosis to the EJV. The free flap was harvested and anastomosed to the recipient vessels. The flap demonstrated good perfusion, and the serratus fascia was placed directly on the cranioplasty, measuring 7 cm × 5 cm ( Fig. 2A ). The latissimus flap was then draped over the serratus fascia and the rest of the defect, creating dual-layer vascularized coverage over the cranioplasty site ( Fig. 2B ). A full-thickness skin graft from the latissimus donor site was placed over the cranioplasty site. A small split-thickness skin graft was utilized to cover the exposed portion of the peripedicle adipose tissue in the preauricular region.

Postoperatively, the patient recovered without complications, and the chimeric flap and skin grafts healed well at approximately 1-year follow-up ( Fig. 1D – E ). Informed consent was provided, and the patient agreed to the use of their photos and medical information as it pertained to their diagnosis and reconstruction for this manuscript.

## Discussion

We describe a chimeric free flap for *multilayered* coverage following a large scalp skin excision and cranioplasty. The dynamic subscapular vascular system has been exploited for coverage of large scalp defects, and this case presents yet another application of this donor site. 5 The viability of the serratus anterior fascia flap has been extensively demonstrated in the hand literature, where it is often used to reconstruct defects in the dorsum, facilitating improved tendon gliding. 9 10 Historical anatomic studies of the subscapular system demonstrate that this fascia can be reliably harvested from the serratus branch of the thoracodorsal artery. 11 Harvesting the fascia does not add morbidity, providing that the long thoracic nerve is spared, as performed in this case. 9

Literature describing the reconstruction of composite scalp and cranium defects has indicated that implant exposure is the most common late complication (10–16%). 6 7 Flap atrophy is the most frequent cause (10%), and the authors suggest that increased soft tissue bulk would be beneficial. 7 However, while bulkier flaps can be utilized, they often result in noticeable aesthetic deformities. Employing multiple layers of vascularized soft tissue coverage, including a layer of serratus fascia, may offer enhanced outcomes compared to a muscle–skin graft only reconstruction.

Furthermore, it is important to note that patients undergoing craniotomy frequently require reoperation for their intracranial process and/or cranioplasty complications. 12 We conjecture that the area of flap overlaying the cranioplasty would be easier to re-elevate because of the underlying serratus fascia. This idea has been mentioned in the lower extremity reconstruction literature when comparing fasciocutaneous to muscle-only flaps. In our case, this would be advantageous if the patient required re-elevation of the flap or cranioplasty in the future. 13 This concept has further been validated in the hand literature, with multiple reports utilizing serratus fascia over extensor tendons to allow for tendon gliding and possible future tendon reconstruction. 10

To our knowledge, this is the first reported case utilizing a chimeric latissimus dorsi and serratus anterior fascia-only flap for single-stage reconstruction of a large composite scalp and calvarial defect. An in-depth literature review of scalp reconstruction utilizing chimeric free flaps produced 14 articles ( Table 1 ). There is extensive literature discussing chimeric flaps incorporating serratus anterior *muscle* or *myo-osseous* components, 1 3 4 5 6 7 14 15 16 17 18 19 20 where the additional soft tissue coverage offered by the serratus muscle is utilized for defects with extensive surface area. However, there is no discussion of utilizing a vascularized fascial layer to allow for ease of potential flap elevation in the future. Our literature review showed that there was no significant association between flap type and long-term complication rates, with an overall recommendation to offer the simplest and most reliable reconstruction whenever possible. 2 6 7 16

**Table 1:** Cranial reconstruction flaps and surgical outcomes

Study	Number of flaps ( *N* )	Mean age (years)	Flap type	Chimeric flap used (yes/no)	SA fascia used (yes/no)	Etiology	Flap survival	Major complication	Minor complication
Berli et al 1	7	63.1	Customized cranial implant + pericranial flap	No	No	Neoplasm	100%	None	Dural tear (1)
Hussussian and Reece 3	32	55	LD, SA + rib, TRAM, VRAM, Scapular fasciocutaneous	Yes; SA muscle + rib	No	Neoplasm	100%	Vein thrombosis (2)	Wound complications
Serra et al 4	1	52	SA myocutaneous + LD myocutaneous	Yes; SA myocutaneous + LD myocutaneous	Yes	Prior surgery-related defect	100%	None	None
Liu et al 5	8	38.7	Staggered LD free flaps	No	No	Trauma	100%	None	Flap congestion (1)
Chao et al 6	125	62.4	LD (main), SA, others	No	No	Cancer	97.8%	Respiratory failure, thrombosis	21% periop, 11.6% late complications
Slijepcevic et al 7	82	NR	LD, RA, ALT, SA/rectus	No	No	Mixed	87%	Flap atrophy (5%)	Implant exposure (16%)
Seitz et al 14	5	62	LD + rib intercostal	No	No	Mixed	100%	Arterial thrombosis	Wound debridement (2)
Lipa and Butler 15	5	64	LD	No	No	Malignancy	100%	None	Seroma, partial graft loss
Othman et al 16	16	70.5	ALT, LD, RFFF	No	No	Malignancy	83.3%	Flap loss (3)	NR
Sweeny et al 17	47	58	LD, RA, RFFF	No	No	Malignancy	96.6%	Flap failure (2)	Hematoma, dehiscence
Lee et al 18	1	22	LD + SA + rib	Yes; LD + SA + rib	Yes	Trauma	100%	None	None
Bassiri Gharb et al 19	56	10	LD + rib myocutaneous	Yes; LD + rib myocutaneous	No	Infection, trauma	100%	Hemothorax, hernia	Donor dehiscence (1)
Hong and Lim 20	6	36.5	LD + rib myocutaneous, rib graft	Yes; LD + rib myocutaneous	No	Trauma	100%	None	Hematoma, effusion

Abbreviations: ALT, anterolateral thigh; LD, latissimus dorsi; *N* , number; NR, not reported; RA, rectus abdominis; RFFF, radial forearm free flap; SA, serratus anterior; TRAM, transverse rectus abdominis myocutaneous flap; VRAM, vertical rectus abdominis myocutaneous flap.

Our approach introduces a novel, multilayered reconstructive strategy that leverages the vascularized, thin, and pliable serratus fascia—harvested quickly and without added morbidity—to reinforce cranioplasty coverage and potentially mitigate long-term complications such as flap atrophy and implant exposure. Additionally, this technique may improve ease of re-elevation in cases requiring future neurosurgical access. Unlike staggered reconstructions or chimeric flaps requiring bulkier components, 5 6 18 19 20 our case offers a true single-stage reconstruction using a serratus fascia-based dual-layer construct that minimizes donor site morbidity. Of note, while our 1-year follow-up demonstrates encouraging outcomes, potential longer-term complications can occur years after soft tissue reconstruction.

This case report demonstrates the successful application of a chimeric free flap, combining the latissimus dorsi and serratus anterior fascia, for dual-layer soft tissue coverage for a large scalp skin defect and cranioplasty. This technique presents an additional viable option for complex scalp reconstruction.
